# Comparative analysis of tumor spheroid generation techniques for differential *in vitro* drug toxicity

**DOI:** 10.18632/oncotarget.7659

**Published:** 2016-02-24

**Authors:** Shreya Raghavan, Pooja Mehta, Eric N. Horst, Maria R. Ward, Katelyn R. Rowley, Geeta Mehta

**Affiliations:** ^1^ Department of Materials Science and Engineering, University of Michigan, Ann Arbor, MI, USA; ^2^ Department of Biomedical Engineering, University of Michigan, Ann Arbor, MI, USA; ^3^ Macromolecular Science and Engineering, University of Michigan, Ann Arbor, MI, USA

**Keywords:** ovarian cancer, breast cancer, high throughput, multicellular tumor spheroids, preclinical drug testing

## Abstract

Multicellular tumor spheroids are powerful *in vitro* models to perform preclinical chemosensitivity assays. We compare different methodologies to generate tumor spheroids in terms of resultant spheroid morphology, cellular arrangement and chemosensitivity. We used two cancer cell lines (MCF7 and OVCAR8) to generate spheroids using i) hanging drop array plates; ii) liquid overlay on ultra-low attachment plates; iii) liquid overlay on ultra-low attachment plates with rotating mixing (nutator plates). Analysis of spheroid morphometry indicated that cellular compaction was increased in spheroids generated on nutator and hanging drop array plates. Collagen staining also indicated higher compaction and remodeling in tumor spheroids on nutator and hanging drop arrays compared to conventional liquid overlay. Consequently, spheroids generated on nutator or hanging drop plates had increased chemoresistance to cisplatin treatment (20-60% viability) compared to spheroids on ultra low attachment plates (10-20% viability). Lastly, we used a mathematical model to demonstrate minimal changes in oxygen and cisplatin diffusion within experimentally generated spheroids. Our results demonstrate that *in vitro* methods of tumor spheroid generation result in varied cellular arrangement and chemosensitivity.

## INTRODUCTION

The multicellular tumor spheroid is an excellent *in vitro* model utilized in cancer biology and toxicology [1–3]. Tumor spheroids mimic avascular *in vivo* tumors and present similar diffusional limitations to the mass transfer of oxygen, nutrients and waste. The cell-cell interactions and cell-extracellular matrix interactions in spheroids are also noted to significantly mimic *in vivo* cyto-architectural conditions in a manner which is more physiologically relevant when compared to two-dimensional monolayer cultures of cells. Gene expression profiles of cells grown in the three-dimensional microenvironment better mimic clinical conditions, when compared to monolayer cultures [1, 2, 4]. Improving the predictive potency of *in vitro* drug screens and enabling a stronger clinical efficacy prediction is of prime importance in several cancers, including breast and ovarian cancers [5]. The spheroid model has been an important therapeutic tool for positive selection of novel drug and biologic candidates for several cancers [3].

Several spheroid-generation techniques have been described in literature that can generate uniform spheroids, for high throughput analysis and screening of chemotherapeutic agent sensitivity [4, 6]. The most common and inexpensive method to generate spheroids involves liquid overlay, where cells plated onto non-adherent surfaces self-assemble in the absence of an adhesive substrate into three-dimensional structures [7]. Conventional hanging drop culture to generate spheroids is also a popular method, but involve tedious maintenance making long-term analysis of spheroids cumbersome [8]. Other techniques utilize shear forces or microgravity to maintain cells in suspension, but require specialized equipment [8].

We recently demonstrated the stable incorporation and formation of ovarian cancer spheroids with as few as 10 cells using hanging drop array plate [9]. Spheroids generated on our platform had significant three-dimensional presence, and demonstrated a higher degree of resistance to conventional chemotherapy agent, cisplatin. Moreover, this platform is conducive to multiplexed analysis, high-throughput amenable and allowed for long-term spheroid culture with minimal maintenance [9].

In this study, we undertook a comparison of spheroids generated with the hanging drop array method, with spheroids generated on the commercially available ultra-low attachment liquid overlay surface, the Nunclon™ Sphera™. We hypothesized that adding a gentle rotating mixer step (using a nutator) will enhance the aggregation and formation of spheroids on the ultra low-attachment plates. The use of nutators to enhance cell aggregation has been described previously [10]. We compared these two methodologies, namely liquid overlay on ultra-low attachment plates with and without rotating mixing (nutation), with our established hanging drop array platform. We evaluated resultant spheroid morphology, architecture, extracellular matrix deposition, and response to chemotherapy agent, cisplatin. Lastly, we performed mathematical analyses based on our experimental data to demonstrate diffusional gradients for oxygen and cisplatin established in spheroids generated using all three methods.

While spheroids are valuable *in vitro* tools in pre-clinical chemosensitivity screens, not all spheroid generation techniques are equivalent [8]. Systematic evaluation of spheroid generating methodologies will be useful tool in utilizing the appropriate method for specific spheroid-based functional and target analyses.

## RESULTS

### Formation and morphometry of MCF7 spheroids

**Figure 1:**
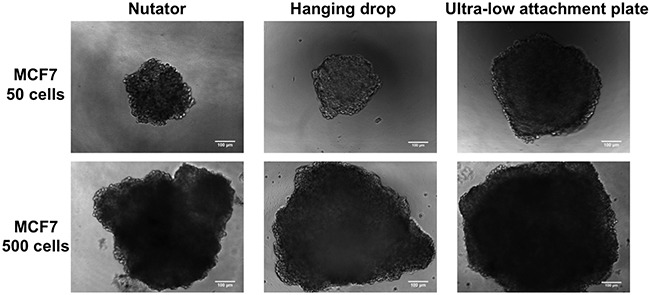
Phase contrast micrographs of MCF7 spheroids at Day 7 generated on hanging drop array plates, ultra-low attachment plates and ultra-low attachment plates with a 48 hour nutation period Visually, spheroids generated from 500 cells were bigger in size than spheroids generated from 50 cells. Sizes of spheroids within the same cell density also varied depending on the method of manufacture. Scale bar = 100μm.

**Figure 2:**
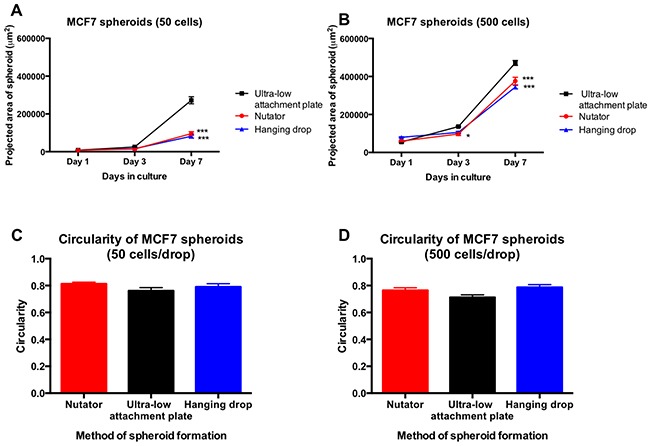
Projected area and circularity of breast cancer spheroids generated on the hanging drop array plates, ultra-low attachment plates with or without nutation **A.** MCF7 spheroids initiated with 50 cells/drop significantly differed (***p<0.0001, one-way ANOVA) in projected area at Day 7 between the three methods of spheroid generation. **B.** MCF7 spheroids initiated with 500 cells/drop demonstrated significant differences (***p<0.0001, one-way ANOVA) beginning at Day 3 between hanging drop, nutator and ultra-low attachment plates. **C, D.** Circularity measurements obtained from spheroids indicated circular morphology of spheroids with values ranging from 0.7-0.9. No significant differences were observed between the different methods of spheroid generation.

MCF7 cells started aggregating on Day 1 in all three platforms used for spheroid generation. Two starting cell-seeding densities were used, namely, 50 cells/drop and 500 cells/drop, and the resulting spheroids were imaged starting Day 1, and followed up until Day 7 in culture. Phase contrast images obtained at Day 7 for MCF7 cells are shown in Figure 1. Calibrated images were used to measure projected area as a measure of spheroid size using Image J. At Day 1, projected area of aggregated MCF7 cells varied non-significantly (7,820±311 μm^2^ to 9,347±585 μm^2^ for 50 cells/drop and 55,164 ± 4,125 to 79,694±3,293 μm^2^ for 500 cells/drop; Figure 2A, 2B) between the three different methods of spheroid generation. The size of spheroids increased with time in culture, as the cells proliferated. At Day 3, MCF7 spheroids generated with the nutator or hanging drop were significantly smaller (*p<0.05, one-way ANOVA, Figure 2B) than spheroids generated on ultra-low attachment plates without nutation, indicating early compaction of cells into a tighter spheroid.

At Day 7, regardless of initial cell seeding density, spheroids generated on hanging drop or nutator plates were significantly smaller (***p<0.0001, one-way ANOVA, Figure 2A, 2B) than spheroids generated on ultra-low attachment plates (compare 81,968 μm^2^ on hanging drop array plates to 272,492 μm^2^ on ultra-low attachment plates for 50 cells/drop MCF7 spheroids). Circularity of spheroids was calculated using Image J from phase contrast images. Circularity data indicated that regardless of method of spheroid generation, circularity varied non-significantly between 0.76 and 0.81 for MCF7 spheroids.

### Chemosensitivity of MCF7 spheroids following cisplatin treatment

**Figure 3:**
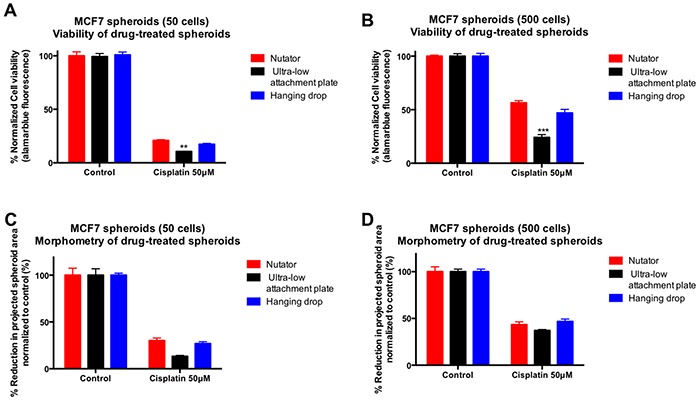
Effect of cisplatin treatment on viability and projected area of breast cancer spheroids following cisplatin treatment **A, B.** MCF7 spheroids initiated with 50 cells/drop demonstrated reduced viability in response to cisplatin treatment. However, cisplatin sensitivity was significantly different depending on the method of spheroid manufacture, i.e. spheroids generated on hanging drop or nutator plates were significantly more chemoresistant to cisplatin (17-20% viable in 50 cells/drop; 46-56% viable in 500 cells/drop) compared to spheroids generated on ultra-low attachment plates (10% viable in 50 cells/drop; 24% in 500 cells/drop). Viability within spheroids was measured using alamarblue fluorescence and normalized to untreated control spheroids generated on the same platform. **C, D.** Morphometry on untreated and cisplatin-treated spheroids indicated a reduction in spheroid projected area with cisplatin treatment. The extent of reduction in projected area was significantly smaller in spheroids from hanging drop arrays or nutator plates, compared to spheroids from ultra-low attachment plates.

MCF7 spheroids generated on ultra-low attachment plates with or without nutation, and spheroids generated on hanging drop array plates were treated with 50μM cisplatin. Viability was determined using alamarblue fluorescence, and normalized to control untreated spheroids. Cisplatin-sensitivity of spheroids varied depending on the method of spheroid generation. Spheroids generated on hanging drop array plates and nutator plates were significantly more chemoresistant to cisplatin (Figure 3A, 3B) compared to spheroids generated on ultra-low attachment plates. Reduction in projected area of spheroids was quantified as a percentage change of drug-treated spheroids compared to untreated control spheroids. Cisplatin-treated spheroids generated on hanging drop array plates or nutator plates demonstrated a 26-30% (50 cells/drop) or 43-46% (500 cells/drop) reduction in projected area compared to 13.27% (50 cells/drop) or 37.17% (500 cells/drop) observed in ultra-low attachment plate generated spheroids (Figure 3C, 3D). Cisplatin treatment of MCF7 spheroids on the ultra low attachment plate resulted in significantly lower cell viability in both 50 cells/drop and 500 cells/drop spheroids (**p<0.001 for 50 cells/drop and ***p<0.0001 for 50 cells/drop, one-way ANOVA, Figure 3A, 3B)

### Formation and morphometry of OVCAR8 spheroids

**Figure 4:**
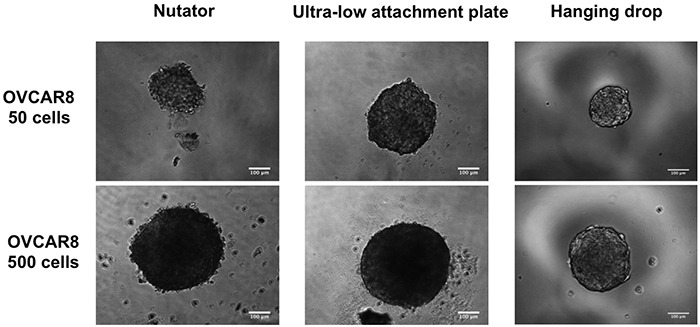
Phase contrast micrographs of OVCAR8 spheroids initiated with 50- or 500 cells/drop on the three platforms Spheroids were generated on hanging drop arrays, ultra-low attachment plates or ultra-low attachment plates with a 48 hour nutation period. Scale bar = 100μm.

**Figure 5:**
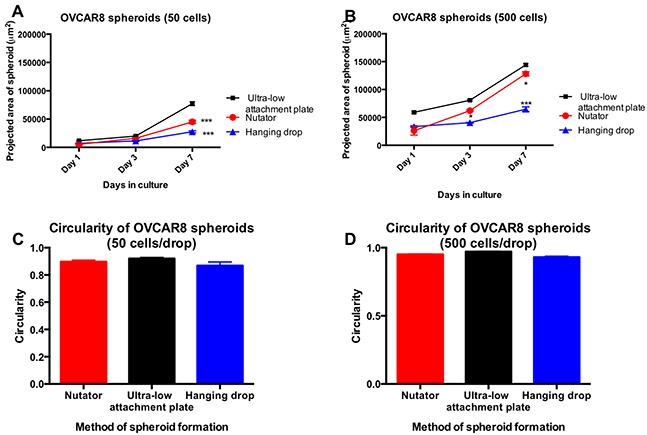
Projected area and circularity of ovarian cancer spheroids generated on the hanging drop array plates, ultra-low attachment plates with or without nutation **A.** OVCAR8 spheroids initiated with 50 cells/drop were significantly smaller (***p<0.0001, one-way ANOVA, compared to ultra-low attachment plate) in projected area when generated either on hanging drop arrays or ultra-low attachment plates on the nutator. **B.** For spheroids initiated with 500 cells/drop, the difference was significant starting from Day 3 (*p<0.05, one-way ANOVA), and remained significantly smaller at Day 7 (***p<0.0001, one-way ANOVA). **C, D.** Circularity of OVCAR8 spheroids did not change significantly depending on the method of spheroid generation, and varied between 0.86 and 0.95.

Similar to MCF7 cells, spheroids were initiated from 50 or 500 cells from the ovarian carcinoma cell line, OVCAR8. These cells also began aggregation at Day 1 following plating, and continued to proliferate and aggregate into spheroids. Live cell microscopy was used to follow up spheroid formation from Day 1 through Day 7. Phase contrast micrographs of OVCAR8 spheroids generated on the three platforms are shown at Day 7, in Figure 4. Similar to MCF7 spheroids, OVCAR8 spheroids generated on the hanging drop array plates or ultra-low attachment plates with nutation had smaller projected areas compared to OVCAR8 spheroids on ultra-low attachment plates by themselves (Figure 5A). At Day7, OVCAR8 spheroids initiated with 50 cells/drop varied in projected area from 77,198±3,521μm^2^ (ultra-low attachment plate) to 45,031±3,043μm^2^ (nutator) to 27,595±1,899μm^2^ (hanging drop).

OVCAR8 spheroids initiated with 500 cells/drop had significant differences in projected area starting Day 3, as the 48-hour nutation period ended (Figure 5B). Spheroids generated on the nutator had average projected areas ranging from 144,082±2,538μm^2^ (ultra-low attachment plate) to 128,085±3,850μm^2^ (nutator) to 64,722±4,186μm^2^ (hanging drop).

Regardless of the method of manufacture, circularity measured from 2D projected images and calculated on Image J, was not different amongst spheroids. Circularity of OVCAR8 spheroids varied non-significantly between 0.86 and 0.97.

### Chemosensitivity of OVCAR8 spheroids following cisplatin treatment

**Figure 6:**
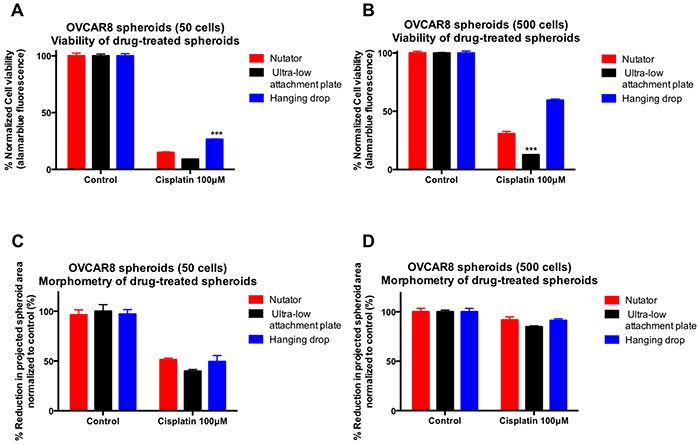
Effect of cisplatin treatment on viability and projected area of ovarian cancer spheroids following cisplatin treatment **A, B.** OVCAR8 spheroids generated on hanging drop arrays were significantly more chemoresistant to cisplatin (26% viable in 50 cells/drop; 59% viable in 500 cells/drop) compared to spheroids generated on ultra-low attachment plates with or without nutation (8-14% viable in 50 cells/drop; 12-30% viable in 500 cells/drop). Viability within spheroids was measured using alamarblue fluorescence and normalized to untreated control spheroids generated on the same platform. **C, D.** Similarly, reduction in projected area and size of cisplatin-treated spheroids was most dramatic in spheroids generated on ultra-low attachment plates, owing to higher sensitivity to cisplatin.

OVCAR8 spheroids were treated with the chemotherapeutic agent, cisplatin. Viability of cisplatin-treated spheroids was measured using alamarblue fluorescence and normalized to control untreated spheroids generated on the same platform. Viability data indicated that in spheroids initiated with both 50- and 500 cells/drop, spheroids generated on the hanging drop array plate were significantly more chemoresistant than spheroids generated on the ultra-low attachment plates with or without nutation (Figure 6A, 6B). In response to 100μM cisplatin, only 8.9±0.2% (50 cells/drop) or 12.6±0.1% (500 cells/drop) remained viable in spheroids generated on ultra-low attachment plates. Conversely, spheroids generated on hanging drop arrays were viable to a much higher extent (26.48±0.4% to 59.53±1%). Similarly, spheroids generated on ultra-low attachment plates with the nutator were also more chemoresistant and more viable following cisplatin treatment, ranging from 14.95±06% (50 cells/drop) to 30.74±2% (500 cells/drop).

Morphometry of images obtained from drug treated spheroids was performed and compared to control untreated spheroids generated on the same platform (Figure 6C, 6D). The sharpest drop in projected area was observed in cisplatin-treated OVCAR8 spheroids generated on ultra-low attachment plates (39.89±1.7% in 50 cells/drop and 84.87±1% in 500 cells/drop). Conversely, projected areas of drug treated spheroids reduced less dramatically and to a much smaller extent when generated on ultra-low attachment plates with nutation or hanging drop arrays (49-51% in 50 cells/drop; 91.2-91.7% in 500 cells/drop).

### Histochemical characterization of MCF7 and OVCAR8 spheroids

**Figure 7:**
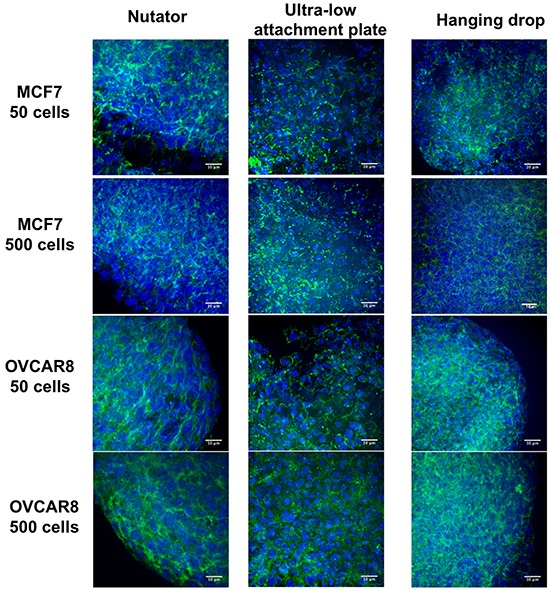
Three-dimensional structure of multicellular breast and ovarian cancer spheroids generated on the three platforms Actin cytoskeletons were stained with AlexaFluor488-conjugated Phalloidin (green), and nuclei were counterstained with DAPI (blue). Cells within spheroids were packed in a compact manner, and cortical actin staining indicated three-dimensionality of all stained spheroids. Scale bar = 10μm.

MCF7 and OVCAR8 spheroids were harvested on a soft bed of agarose, and stained with fluorescently conjugated Phalloidin to visualize cytoskeletal networks and cell-cell interactions (Figure 7). Nuclei were counterstained blue with DAPI. Phalloidin staining on spheroids indicated cortical actin staining, typical of three-dimensional cultures, as opposed to the diffuse staining observed in 2D cultures of cells. Moreover, dense packing of nuclei and interwoven cortical actin staining indicated three-dimensional morphology as well as cell-cell interaction in spheroids.

**Figure 8:**
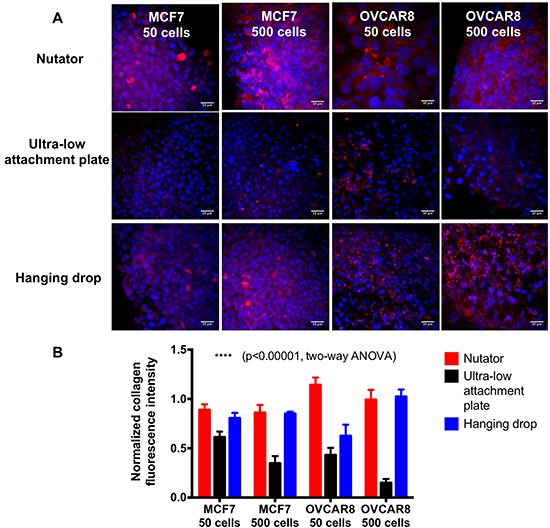
Extracellular matrix protein deposition in the multicellular breast and ovarian cancer spheroids generated on the three platforms Collagen I was stained and visualized using a TRITC-conjugated secondary antibody, and red fluorescence indicated the presence of collagen within the spheroids. Nuclei were counterstained with DAPI (blue). Collagen staining was visually more abundant in spheroids generated on ultra-low attachment plates with nutator, compared to ultra-low attachment plates without nutator. Collagen staining was also abundant on spheroids generated on hanging drop arrays, but sparse on spheroids from ultra-low attachment plates by themselves. Collagen fluorescence was quantified by imaging spheroids generated using the three different methods at identical gain and exposure settings on a confocal microscope. Quantification of collagen staining using fluorometry indicated that collagen expression in nutator plates and hanging drop array plates were comparable, but significantly reduced in spheroids on ultra-low attachment plates. Scale bar=10μm.

The extracellular matrix protein, collagen type I, was visualized using red fluorescence (Figure 8). Collagen type I staining was abundant in the intercellular space between cells as observed by the red fluorescence, indicating the remodeling of the spheroid microenvironment by the cells. Fluorescence microscopy was carried out at identical gain and exposure settings for collagen type I and DAPI between spheroids generated on nutator, ultra-low attachment plates and hanging drop arrays to allow for fluorometric comparisons. Further, collagen type I fluorescence was normalized to DAPI fluorescence. Quantification of normalized collagen type I using fluorometry indicated that spheroids generated on ultra-low attachment plates had a lower amount of collagen type I (0.15 to 0.61 AU) compared to spheroids generated on nutator (0.88 to 1.14 AU) or hanging drop arrays (0.62 to 1.02 AU; Figure 8B).

### Mathematical modeling of diffusion of oxygen and cisplatin in experimental spheroids of varying circularity

**Figure 9:**
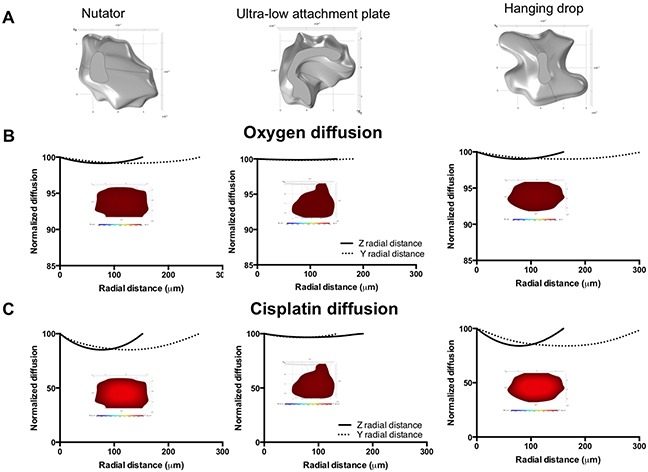
Diffusion profiles of oxygen and diffusion in 500 cells/drop breast cancer experimental spheroids as a function of platform used for spheroid generation **A.** SolidWorks models of MCF7 spheroids (500 cells/drop) generated on the nutator, ultra-low attachment plates or hanging drop arrays. **B.** Oxygen diffusion gradient plots and diffusion plots on experimental spheroids. **C.** Cisplatin diffusion plots and gradients on experimental MCF7 spheroids. Gradient plot color scale for oxygen: 100% (red) to 30% (blue); Gradient plot color scale for cisplatin: 100% (red) to 0% (blue).

**Figure 10:**
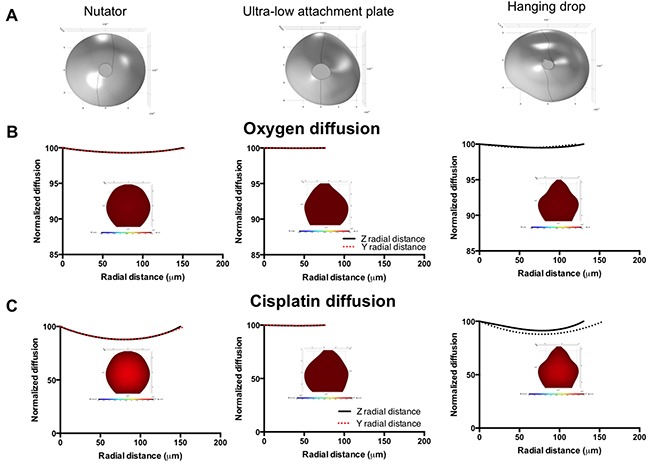
Diffusion profiles of oxygen and diffusion in 500 cells/drop ovarian cancer experimental spheroids as a function of platform used for spheroid generation **A.** SolidWorks models of OVCAR8 spheroids (500 cells/drop) generated on the nutator, ultra-low attachment plates or hanging drop array plates. **B.** Oxygen diffusion gradient plots and diffusion plots on experimental spheroids. **C.** Cisplatin diffusion plots and gradients on experimental OVCAR8 spheroids. Gradient plot color scale for oxygen: 100% (red) to 30% (blue); Gradient plot color scale for cisplatin: 100% (red) to 0% (blue).

In order to discern the effect of diffusion due to differences in spheroid generation techniques, we utilized experimental spheroids (500 cells/drop) to generate three-dimensional models on SolidWorks (Figure 9A; MCF7 spheroids; Figure 10A; OVCAR8 spheroids). These models were imported into a COMSOL module for ‘transport of diluted species’.

Figure 9B demonstrates changes in oxygen diffusion in the y and z depth axes of MCF7 spheroids generated on nutator, ultra-low attachment or hanging drop array plates. Model data demonstrates that oxygen diffusion dropped less than 0.7% in either of the depth directions of all MCF7 spheroids at their core, with the lowest drop in oxygen amounting to 99.3% (Figure 9B). Cisplatin concentration gradients were significantly sharper on spheroids generated on nutator or hanging drop plates dropping to 85.19% and 83.96% of the original concentration respectively. In contrast, cisplatin concentration only dropped to 96.85% in MCF7 spheroids generated on ultra low attachment plates (Figure 9C). Graphical plots representing change in oxygen and cisplatin concentration in MCF7 spheroids along with representative gradient plots are shown in Figure 9B, 9C.

In OVCAR8 spheroids, oxygen drop was less than 0.7% in the z or y directions of all spheroids irrespective of method of spheroid generation (Figure 10B). Similar to MCF7 spheroids, cisplatin concentrations varied between the spheroids dropping to 87.84% (nutator), 91.25% (hanging drop) and 99.48% (ultra-low attachment plate), as shown in Figure 10C. Cisplatin diffusion data indicates that the change in cisplatin concentration can account partially for the differences observed in cisplatin sensitivity in spheroids generated on nutator plates, hanging drop arrays or ultra-low attachment plates.

## DISCUSSION

Multicellular tumor spheroids are excellent *in vitro* models of avascular tumors for understanding cancer biology, as well as, for preclinical drug studies [8]. Several instances of external mechanical forces used to generate spheroids exist, but are not conducive to visualization of spheroid formation, and achieving uniform spheroid shape and sizes and other morphological characteristics essential for drug screening [4, 5, 11, 12, 16, 17].

In this study, we compared three different methods of spheroid-generation to one another in terms of morphology, chemosensitivity and extracellular matrix deposition. Using a commercially available ultra-low attachment plate, we added a 48-hour rotating mixer (nutation) step in order to enhance the aggregation of cells while preventing their gravity-induced precipitation. The Nunclon™ Sphera™ 96-well U-bottom plate was chosen due to commercial availability, and its widely validated use in generation of spheroids from several cancer cell lines [13–15].

We demonstrated spheroid formation using two cell types, the breast cancer metastatic cell line MCF7 and the ovarian adenocarcinoma cell line OVCAR8. We generated spheroids on hanging drop arrays, ultra-low attachment plates or ultra-low attachment plates with a 48-hour nutation (nutator plates). Our results indicated that spheroid size vastly differed as a function of two variables; initiating cell density determined spheroid size (Figure 2A, 2B; Figure 5A, 5B), in line with our previous observations on small cell number ovarian cancer spheroids [9]. The second contributor to variation in spheroid size was the method of spheroid generation. Both OVCAR8 and MCF7 spheroids were significantly smaller when generated either on nutator plates or hanging drop arrays, indicating that nutator plates promote higher cellular aggregation and compaction compared to ultra-low attachment plates, owing to the external forces the cells experience from the 48-hour nutation period. Since hanging drop arrays are true representatives of non-adherent culture due to the absence of a substratum itself, cellular aggregation was promoted based on gravity [18]. Since ultra-low attachment plates use polymeric surface coatings to hinder cellular adhesion, we initially expected spheroid circularity to be significantly lowered compared to non-adherent hanging drop cultures [6]. However, no significant difference in circularity was observed in spheroids generated on all three platforms, indicating that all three surfaces were sufficiently non-adherent and promoted spheroid formation. Cortical actin staining confirmed the three-dimensional nature of the spheroids [19].

We demonstrated an *in vitro* chemosensitivity assay using a conventional chemotherapeutic agent, cisplatin, to ascertain if a difference in size resulted in a difference in drug sensitivity. When comparing the untreated control spheroids generated with the three methods, there were no significant differences in the cell viability after 7 days of spheroid growth. Following 72 hours of cisplatin treatment, both MCF7 and OVCAR8 spheroids generated on either nutator plates or hanging drop arrays, were more chemoresistant to cisplatin compared to spheroids generated on ultra-low attachment plates. Comparing cisplatin-sensitivity across different platforms, smaller spheroids on the nutator or hanging drop array were significantly more chemoresistant. This can be correlated with the higher extracellular matrix content observed in spheroids generated on the nutator and hanging drop array plates compared to spheroids generated on ultra-low attachment plates by themselves. Mathematical modeling also indicated changes in cisplatin diffusion through experimental spheroids, indicating that other than the extracellular matrix deposition impeding diffusion, observed chemoresistance could additionally be mediated through cell-cell and cell-ECM adhesions reported by several others in a variety of cancers [20–22].

Lastly, we used diffusion-based mathematical modeling to demonstrate changes in oxygen and cisplatin diffusion in asymmetric solid models of experimentally derived spheroids. We validated our model by modeling ideal spheroid geometry with a diameter of 2500 μm (Supplementary Figure S1), where a conventional hypoxic core is observed for oxygen. We then modeled ideal spheroid and ellipsoid shapes with a 500μm diameter, using cellular compaction factors derived from experimental values of projected area, and collagen deposition (Supplementary Figure S2, Supplementary Figure S3). It is not surprising that we do not see the conventional hypoxic core often observed during oxygen diffusion modeling in spheroids, owing to the small size of our spheroid models (250μm and 500μm) [23, 24]. The predictive ability of our model was bolstered by the use of compaction factors that were experimentally derived based on both projected area/size of spheroids, as well as, collagen deposition. Both these factors were identified based on quantification performed in Figures 2, 4, 8B to vary oxygen and cisplatin consumption/uptake rates and diffusional constants. Such variation of cellular consumption rates has been performed previously by Leung *et al.* in order to account for cellular compaction [25]. Even though the mathematical model we used to identify diffusion was simplistic, we used solid models from experimentally derived spheroids generated on different platforms with experimentally derived geometry, as opposed to performing mathematical analysis on theoretical spheroid shapes [26–28]. Our modeling data also demonstrated that cisplatin gradients could partially account for heightened cisplatin chemosensitivity in spheroids generated on ultra-low attachment plates when compared to spheroids generated on nutator or hanging drop array plates. Our results underline the significance of the method of spheroid generation on important aspects of spheroid geometry, cellular organization within spheroids, diffusion of nutrients, drugs and metabolites within spheroids and drug toxicity. Different technologies have been developed for generating multicellular tumor spheroids. While spheroids are valuable *in vitro* tools in pre-clinical chemosensitivity screens, not all spheroid generation techniques are equivalent. Our work illustrates that the technique used for generating spheroids has a significant impact on the resultant cellular phenotypes.

## MATERIALS AND METHODS

### Materials

All cell culture reagents and supplements were purchased from Life Technologies (Carlsbad, CA), unless specified otherwise. Growth medium was RPMI 1640 supplemented with 10% fetal bovine serum and 1X antibiotics/antimycotics. 96-well U-bottom Nunclon ™ Sphera™ microplates were used as ultra-low attachment plates. Alexafluor 488-Phalloidin and DAPI were purchased from Life Technologies, and rabbit polyclonal Collagen I antibody was obtained from Abcam. The breast cancer cell line, MCF7, was obtained from American Type Culture Collection (Manassas, VA). The ovarian cancer cell line, NIH:OVCAR8, was a generous gift from Dr. Nouri Neamati (University of Michigan). Cisplatin was purchased from Sigma Aldrich (St. Louis, MO). Hanging drop array plates were purchased from XCentric Mold and Engineering (Clinton Twp, MI).

### Formation of breast and ovarian cancer spheroids

MCF7 or OVCAR8 cells were cultured in growth media until 70% confluency was reached. Cells were trypsinized as per a regular passage and counted using a hemocytometer. Cell dilutions were adjusted in such a way that a 20-50μl volume contained either 50 or 500 cells. Three methods were utilized to generate spheroids: 1) hanging drop array plates; 2) ultra-low attachment plates; and 3) ultra-low attachment plates with a 48-hour nutation period (nutator plates).

For the hanging drop array plates, 50 cells/drop and 500 cells/drop of MCF7 and OVCAR8 spheroids were plated as described previously [9]. In a similar manner, 50μl drops containing 50 cells/drop or 500 cells/drop of MCF7 or OVCAR8 cells were plated into ultra-low attachment plates. At least 20 replicates were plated of each cell type for each cell density on each plate. 3-5 biological replicates were carried out of the same experiments.

For mechanical agitation facilitated spheroid formation, ultra-low attachment plate was placed on a fixed speed nutator (Fisher Scientific, 260100) housed inside a CO_2_ incubator for 48 hours. Following the 48-hour nutation period, the plate remained in static culture upto Day 7. All plates were incubated for 7 days following initial plating. Medium was supplemented every alternate day to maintain proliferation and viability in all plates.

### Observation of spheroid formation and morphometry

Hanging drop array plates, ultra-low attachment plates and nutator plates were removed periodically for microscopic observation of spheroid formation as described previously [9]. Live cell phase contrast microscopy was performed using a calibrated inverted microscope (Olympus IX81, Japan, equipped with an ORCA R2 cooled CCD camera and CellSens software). 3-5 individual plates of each condition were imaged, and 3-5 images were obtained from each plate to obtain morphometric data. Calibrated images were exported to NIH Image J (National Institutes of Health, Bethesda MA) for morphometric analysis. The polygon tool was used to outline spheroids and projected area and circularity measurements were obtained. Circularity is measured as (4Π×[Area])/[Perimeter]^2^, and ranges from 0 for infinitely elongated polygon to 1 for perfect circle.

### Viability of spheroids after chemotherapeutic agent treatment

Spheroids generated on hanging drop array plates, ultra-low attachment plates or nutator plates were allowed to aggregate and self-organize for 4 days. Following 4 days of incubation, spheroids were either treated with 50μM cisplatin or left untreated for 72 hours. Alamarblue dye (Life Technologies, Carlsbad, CA) was added in a 1/10 dilution to spheroids to assess viability as described previously [9]. Alamarblue fluorescence readings were obtained at 530nm/590nm on a fluorescence plate reader (BioTek Instruments, Winooski VT). Viability (alamarblue fluorescence) of cisplatin-treated spheroids was expressed as a percentage compared to untreated control spheroids. Images of drug-treated spheroids were obtained and morphometric measurements were performed. Reduction in spheroid size with drug-treatment was calculated based on the difference in sizes between untreated control spheroids and cisplatin-treated spheroids, expressed as a percentage.

### Immunohistochemical staining of spheroids

Spheroids were harvested onto a soft bed of 2% agarose on a microscope slide, using 200μl micro-pipets. Care was taken so as not to disturb the spheroids while transferring them to be embedded onto agarose for further immunohistochemical staining. Spheroids were fixed in 4% neutral buffered formalin, blocked with 10% goat serum and mildly permeabilized using 0.1% Triton-X. Alexafluor488-conjugated phalloidin was utilized to visualize actin cytoskeletons in spheroids generated on hanging drop array plates, nutator plates and ultra-low attachment plates. Collagen staining was carried out by a 1-hour incubation with the rabbit polycloncal anti-Collagen type I antibody (Abcam), followed by three washes to remove unbound antibody and a subsequent 1-hour incubation with TRITC-conjugated anti-rabbit secondary antibody. All spheroid samples (nutator, ultra-low attachment plates, and hanging drop arrays) were treated with the same titer for both primary and fluorophore conjugated secondary antibodies. Nuclei were counterstained with DAPI. Fluorescence for nuclei, actin network staining or collagen type I was visualized using a confocal microscope (Olympus IX81, equipped with a Yokogawa CSU-X1 confocal scanning laser unit, Andor iXon x3 CCD camera, and Metamorph 7.8 software). All spheroid samples were imaged at the same gain and exposure setting (800 millisecond) for red TRITC collagen type I fluorescence. The blue DAPI counterstain was also obtained at identical gain and exposure settings (40 millisecond) between samples to visualize nuclei within the spheroids. A range of z-stacks were obtained for each spheroid imaged, and a reconstruction of an XY image was created using Metamorph for Olympus software. All images were obtained on a 10X magnification for fluorometric quantification in order to visualize the entire spheroid. The 3D reconstructions of MCF7 or OVCAR8 spheroids generated on nutator, ultra-low attachment plates or hanging drop arrays were imported into Image J and a histogram was generated to read the mean intensity on the red (collagen type I) and blue (nuclei) channels. The red collagen intensity was normalized to the blue nuclei intensity to obtain normalized collagen intensity per spheroid, to compare spheroids among platforms.

Normalized fluorescence intensity of collagen type I was compared across spheroids generated using the 3 methods and 2 initial cell densities. Multiple confocal images (3-5) were used for the quantification of normalized collagen type I fluorescence intensity.

### Mathematical modeling of oxygen and cisplatin

Mathematical modeling was utilized to determine oxygen and cisplatin gradients within spheroids and to demonstrate the effect of spheroid geometry and aspect ratio on mass transport. Simulations were run on the commercially available finite element package (Comsol v 5.1, Burlington MA). Physiologically relevant 3D asymmetric models were evaluated. Models were based on experimental images obtained on the confocal microscope of MCF7 and OVCAR8 spheroids generated on the hanging drop array, ultra-low attachment plates or nutator plates. First, 3D projections of spheroid z stacks were compiled and visualized using Image J. Geometries were then created using a commercially available 3D software tool (SolidWorks 2014, Waltham, MA). Topographical sketches were drawn using the spinline tool on several different planes to replicate the topography of the 3D projection. These profiles were wrapped using the lofted boss tool creating a solid spheroid for import into Comsol. These models were run through a ‘transport of diluted species’ module incorporating both diffusion and consumption rates available from literature for oxygen and cisplatin [29–33]. The following values were utilized in our models: Oxygen diffusion coefficient *D = 2 × 10^−10^ m*^2^ *s^−1^*; oxygen consumption rate R = −3.09 × 10^−4^ *mol m*^−3^*s^−1^*; initial concentration of oxygen outside of the spheroid C_o_ = 1.25 *mol m^−3^*; Cisplatin diffusion coefficient *D_cis_ = 5 × 10*^−10^*m^2^s^−1^*; Cisplatin consumption rate R_cis_ = −5.383 × *10^−5^ mol m*^−3^ *s^−1^*, initial concentration of Cisplatin outside of the spheroid C_cis_ = 0.005 *mol m^−3^)*. For spheroids generated on the nutator or hanging drop array plates, compaction factors were used based off of the projected area/size of spheroids and expression of collagen type I based on fluorometric quantification compared to ultra-low attachment plates.

### Data analysis

All experiments were repeated with 3-7 biological replicates, with n ≤ 20, in order to carry out statistics. Statistical data was analyzed on GraphPad Prism 5.0 (www.graphpad.com, San Diego CA). All data is represented as mean ± standard error of the mean. Where appropriate, one-way ANOVA was performed to assess statistical significance, with post-hoc Tukey tests for comparison between means. Levels of statistical significance are indicated on the respective graphs where appropriate.

## CONCLUSIONS

Multicellular spheroids have been a mainstay *in vitro* model of tumor biology and drug screening for the last 40 years. Due to their physiological attributes, many different methodologies for spheroid formation have been developed. In our current study, we systematically evaluated three methods to generate breast and ovarian cancer spheroids. We demonstrate that spheroids generated on hanging drop arrays or ultra-low attachment plates with a 48-hour nutation period are significantly more chemoresistant and have significantly higher amounts of extracellular matrix deposition, compared to spheroids generated on ultra-low attachment plates themselves. This is a significant finding when adopting a suitable method of spheroid generation, to improve predictive potency of *in vitro* drug screening assays.

## SUPPLEMENTARY INFORMATION FIGURES


