# Supplementary material for: Comparative analysis of tumor spheroid generation techniques for differential in vitro drug toxicity
Source: Oncotarget. 2016 Feb 24;7(13):16948–61. doi: 10.18632/oncotarget.7659 (PMC4941362; doi:10.18632/oncotarget.7659)
Supplement: Supplementary file 1 [file oncotarget-07-16948-s001.pdf]

## SUPPLEMENTARY INFORMATION

## SUPPLEMENTARY FIGURES

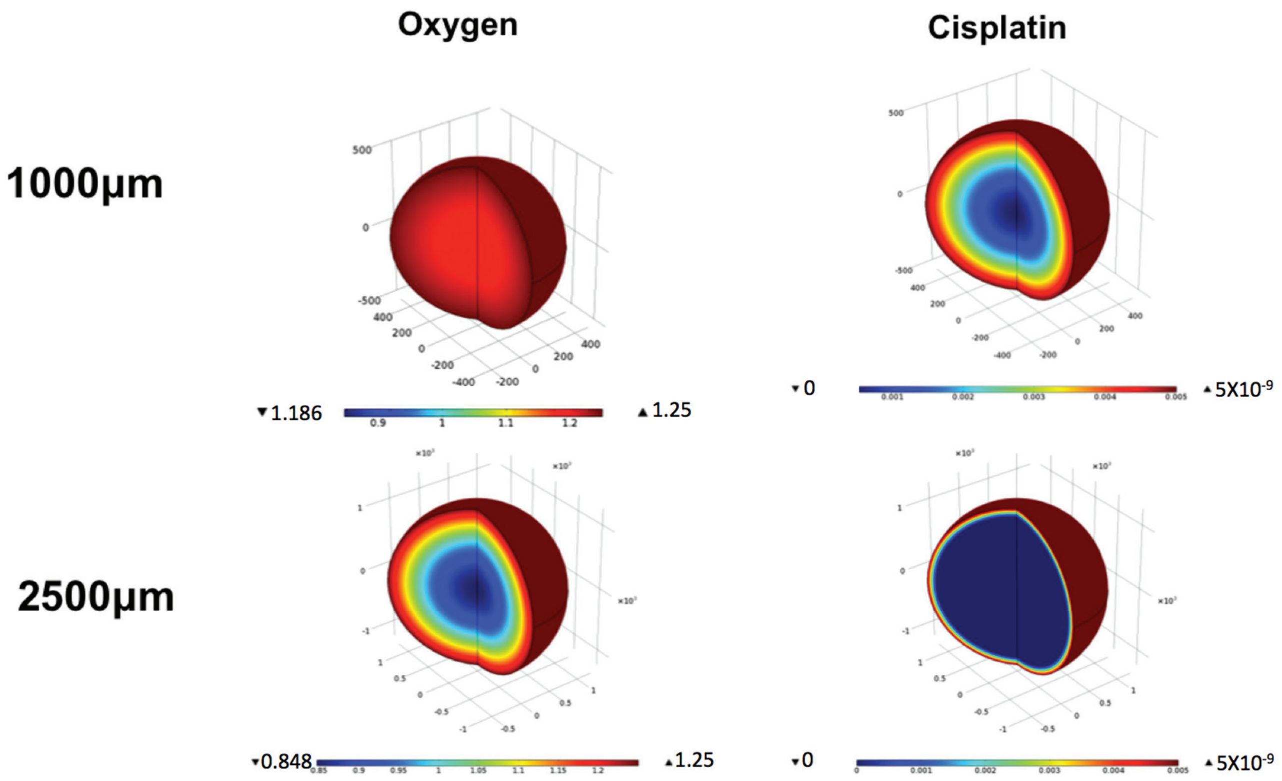


**Supplementary Figure S1: Mathematical modeling of diffusion ofoxygen and cisplatin as a function of ideal spheroid with a diameter of 1000µm or 2500µm.** Gradient plots scaled for oxygen concentration vary from 100% (red) to 68% (blue) and for cisplatin vary from 100% (red) to 0% (blue). For the ideal diameter spheroids of 1000µm diameter, the oxygen concentration only dropped to 95% at its core, while the cisplatin concentration dropped to 10% of its original value at the core. Meanwhile in the 2500µm diameter spheroid, the oxygen concentration at the core dropped to 68% of original oxygen, and for cisplatin concentration dropped to 0% of its original value at the core, indicating that with increasing spheroid sizes, diffusional limitations are more obvious.

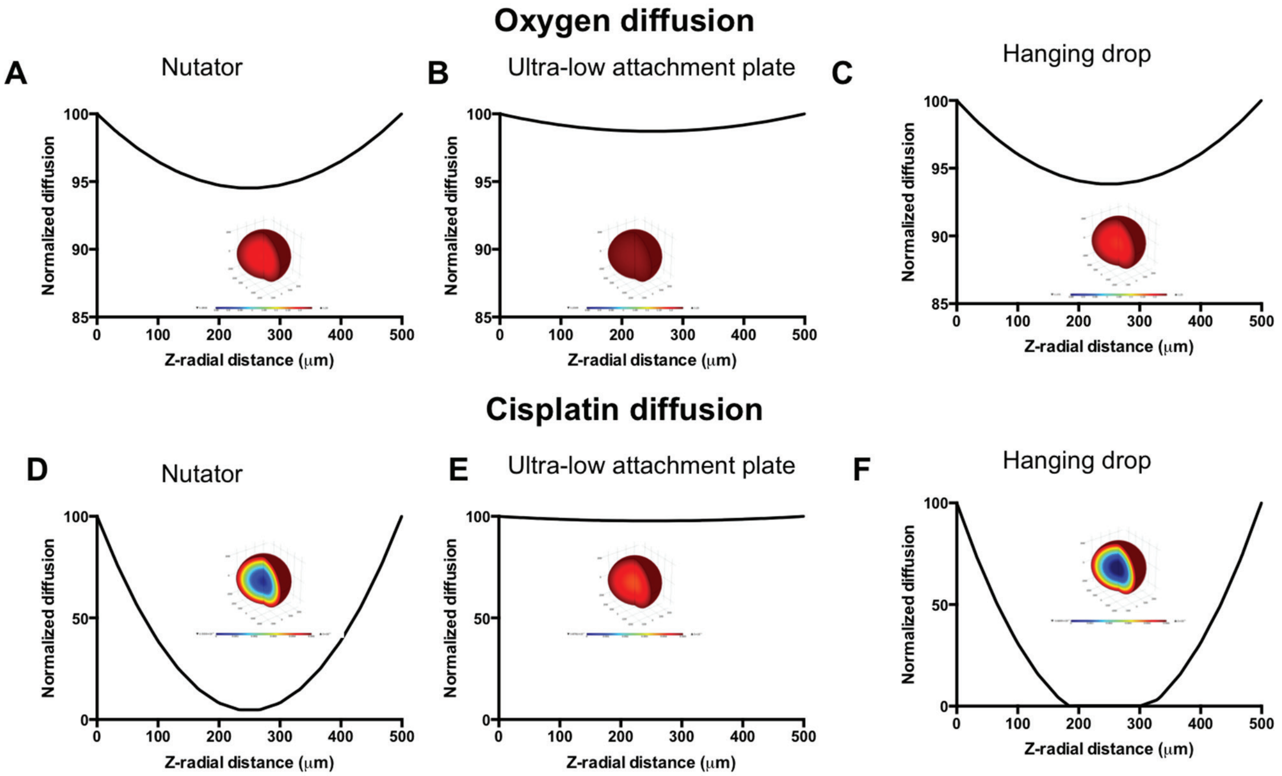


**Supplementary Figure S2: Mathematical modeling of diffusion of oxygen and cisplatin in 500 µm diameter ideal spheroids generated on three different platforms.** Ideal 500µm diameter spheroids were modeled as if generated on nutator plates **A, D.** ultra-low attachment plates **B, E.** or hanging drop array plates **C, F.** Oxygen diffusion plots and gradient plots are shown in **A–C.** and cisplatin plots are shown in **D–F.** Gradient plot scaled for oxygen vary from 100% (red) to 68% (blue), and for cisplatin vary from 100% (red) to 0% (blue). Oxygen and cisplatin consumption rates and diffusion in nutator plates and hanging drop array plates were modified to account for increased cellular compaction owing to smaller size and for increased collagen deposition in spheroids generated on these two platforms.

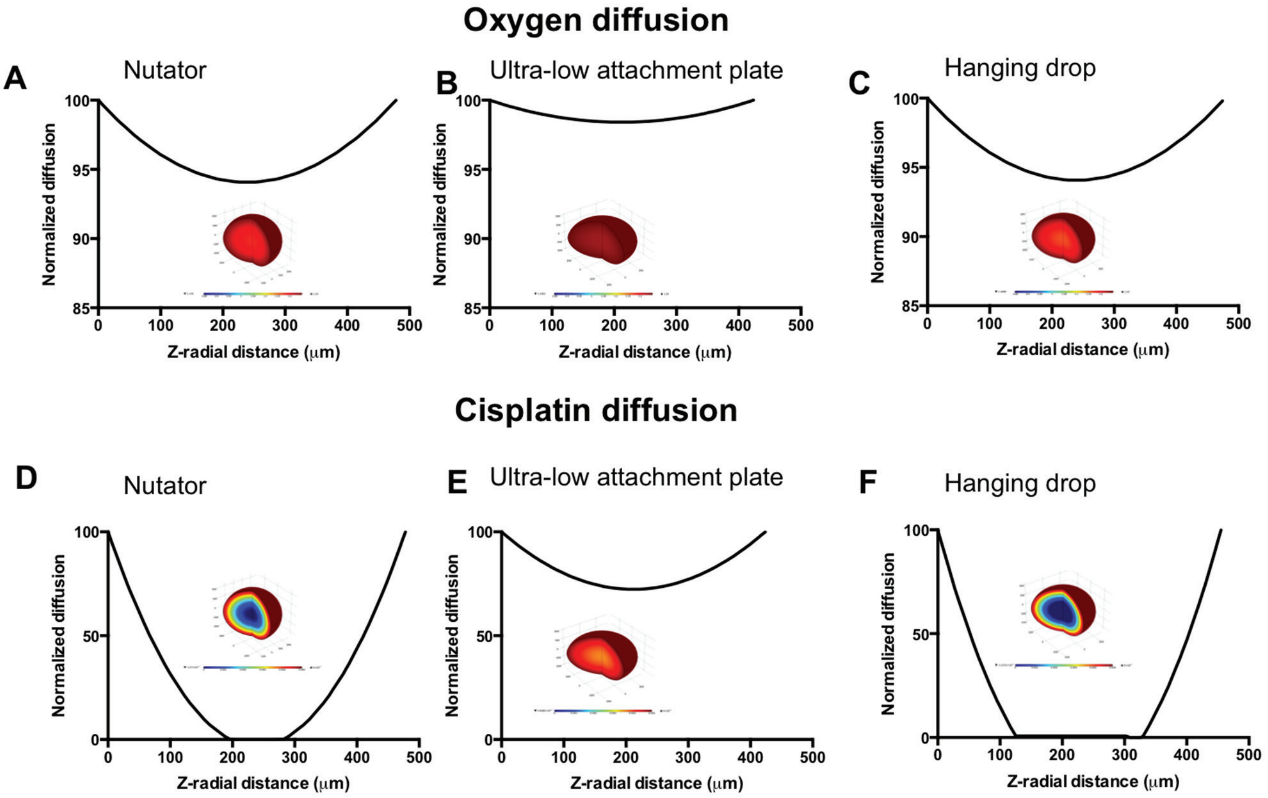


**Supplementary Figure S3: Mathematical modeling of diffusion of oxygen and cisplatin in 500 µm diameter ideal ellipsoids generated on three different platforms.** Ideal 500µm ellipsoids were modeled as if generated on nutator plates **A, D.** ultra-low attachment plates **B, E.** or hanging drop array plates **C, F.** Confocal z-stacks were used to identify aspect ratios for spheroids generated on three-different platforms, and applied to the modeled ellipsoids as Nutator (1.14), Ultra-low attachment plate (1.64) and Hanging drop array plate (1.32). Gradient plot scaled for oxygen vary from 100% (red) to 68% (blue), and for cisplatin vary from 100% (red) to 0% (blue). Oxygen and cisplatin consumption rates and diffusion in nutator plates and hanging drop array plates were modified to account for increased cellular compaction owing to smaller size and for increased collagen deposition in spheroids generated on these two platforms.
